# Neuromorphic Functions of Light in Parity‐Time‐Symmetric Systems

**DOI:** 10.1002/advs.201900771

**Published:** 2019-06-03

**Authors:** Sunkyu Yu, Xianji Piao, Namkyoo Park

**Affiliations:** ^1^ Photonic Systems Laboratory Department of Electrical and Computer Engineering Seoul National University Seoul 08826 Korea

**Keywords:** amplitude death, neuromorphic function, optical nonlinearity, oscillation death, parity‐time symmetry

## Abstract

As an elementary processor of neural networks, a neuron performs exotic dynamic functions, such as bifurcation, repetitive firing, and oscillation quenching. To achieve ultrafast neuromorphic signal processing, the realization of photonic equivalents to neuronal dynamic functions has attracted considerable attention. However, despite the nonconservative nature of neurons due to energy exchange between intra‐ and extra‐cellular regions through ion channels, the critical role of non‐Hermitian physics in the photonic analogy of a neuron has been neglected. Here, a neuromorphic non‐Hermitian photonic system ruled by parity‐time symmetry is presented. For a photonic platform that induces the competition between saturable gain and loss channels, dynamical phases are classified with respect to parity‐time symmetry and stability. In each phase, unique oscillation quenching functions and nonreciprocal oscillations of light fields are revealed as photonic equivalents of neuronal dynamic functions. The proposed photonic system for neuronal functionalities will become a fundamental building block for light‐based neural signal processing.

## 1 Introduction

Ionic mechanisms in a neuron^1^ are governed by the state‐dependent gating of ion channels (Na^+^, K^+^, and leak) and their nonlinear competition satisfying the law of current conservation, which derive unique neuronal functions of bifurcation,^2^, ^3^ repetitive firing,^4^ and oscillation quenching.^5^, ^6^ To achieve neuromorphic signal processing, important clues regarding the analogy of a neuron are therefore in electronic,^7^ spintronic,^8^ and photonic^9^, ^10^, ^11^ implementations of the state‐dependent channel gating and interchannel interactions. In particular, with the advantage of ultrafast signal processing in photonics, the realization of suitable “light” channels that mimic “ion” channels within a neuron has been a key issue of practical importance in the field of neuromorphic photonics.^9^, ^10^, ^11^

For neuromorphic photonic systems, we can envisage the substitution of neuronal ion channels that connect intra‐ and extra‐cellular regions by nonlinear wave amplification and dissipation, due to the similarity between biological and optical state‐dependent dynamics. The study of state‐dependent channels in wave systems is readily found in traditional photonic devices, such as laser with gain saturation^12^ and mode locking^13^ with saturable absorption. The emergence of metamaterial concepts, nanofabrication technologies, and innovative material platforms has also allowed the enhanced strength and tunability of nonlinear amplification or absorption for photonic devices, as shown in coherent amplification in lossy plasmonic metamaterials,^14^ ultrafast pulsed lasing in graphene structures,^15^ or black phosphorus,^16^ and nonlinear activations in photonic deep‐learning circuits.^17^

Non‐Hermitian photonics^18^, ^19^, ^20^ inspired by parity‐time (PT) symmetry^21^ has rejuvenated the utilization of nonlinear wave channels for photonic^22^, ^23^, ^24^ or microwave^25^ functionalities. The concept of PT symmetry^21^ has allowed the access to real observables in non‐Hermitian Hamiltonians. Due to the design flexibility of photonic platforms and the Schrödinger‐like paraxial wave equation for light, photonic structures have been employed as a testbed for examining wave phenomena in PT‐symmetric systems. Early studies have mostly focused on linear wave phenomena near the exceptional point (EP),^20^, ^26^ such as asymmetric^27^ and sensitive^28^ excitations, unidirectional invisibility,^29^ enhanced spin,^30^, ^31^ or orbital angular momentum,^32^ and asymmetric modal conversion,^33^ which have been demonstrated in waveguides,^27^, ^33^ fibers,^29^ metamaterials,^30^, ^31^ and resonators.^28^, ^32^ Recently, a research focus in this field has been extended to the interpretation of nonlinear wave phenomena. The effects of nonlinear wave channels near the EP^26^ have been studied for nonreciprocal transparency from directional resonator excitations,^22^, ^23^ robust power transfer using gain saturation,^25^ and dynamical encircling for polarization conversion which is robust to nonlinear effects.^24^ However, in spite of the inherent non‐Hermitian nature of neurons as shown in energy exchange between intra‐ and extra‐cellular regions of a neuron, an analytical framework to address the critical role of non‐Hermitian phenomena in neuromorphic photonics is still absent.

In this paper, we develop the photonic analogy of a neuron to realize neuromorphic dynamic functions of bifurcation, firing, and quenching by exploiting state‐dependent wave channels that satisfy PT symmetry. Comparing different types of optical nonlinearities to the Hodgkin–Huxley (HH) model,^1^ we show that PT‐symmetric coupled resonators of saturable gain and loss satisfy the criteria of interacting state‐dependent wave channels. We then perform the classification of PT‐symmetric phases in terms of the stability.^2^ The emergence of oscillation quenching functions^5^ in the PT‐symmetric system is demonstrated in relation to PT‐symmetric phases, revealing the transition between amplitude death (AD)^34^ and oscillation death (OD)^35^ across the EP. We also show the directional repetitive firing of light, allowed in the coexistence regime of unbroken and broken PT symmetry. With its multifaceted neuromorphic functions, the proposed PT‐symmetric photonic neuron will serve as a fundamental building block for light‐based artificial neural networks, especially providing functional robustness and directionality.

## 2 Analogy of a Neuron in Nonlinear Photonic Systems

**Figure 1:**
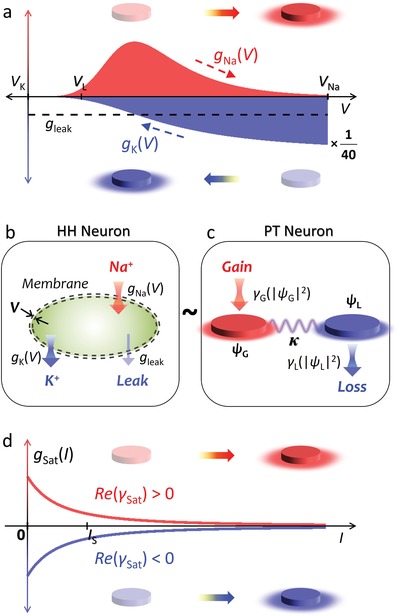
Photonic analogy of a neuron. a) Calculated channel strengths *g*_Na_ (red), *g*_K_ (blue), and *g*_leak_ (black dashed line). *g*_K_(*V*) is divided by 40 for comparison. Upper and lower illustrations of the data plot represent the excitation of gain and loss resonators in (c), respectively, corresponding to the change in membrane potential *V*. b,c) Correspondence between the b) HH neuron and c) its photonic analogy with the PT‐symmetric configuration. The HH neuron is composed of state‐dependent ion channels. The photonic system is composed of saturable gain and loss channels determined by the light intensity |ψ_G_,_L_|^2^ inside each resonator. d) Photonic channel strengths *g*_Sat_(*I*) with different signs of γ_Sat_.

To draw the photonic analogy of a neuron, we examine neuronal ion channels using the HH model.^1^ The dynamics of the membrane potential *V* is described by *dV*/*dt* = ρ_+_(*V*) + ρ_−_(*V*) with ρ_+_(*V*) = *g*_Na_(*V*)·(*V*_Na_−*V*)/*C*_m_ and ρ_−_(*V*) = −[*g*_K_(*V*)·(*V*−*V*_K_) + *g*_leak_(*V* − *V*_leak_)]/*C*_m_, where *C*_m_ is the membrane capacitance; *g*_Na_ and *g*_K_ are the nonlinear conductance of sodium (Na^+^) and potassium (K^+^) ion channels, respectively, and *g*_leak_ is the constant leak conductance; and *V*_Na_, *V*_K_, and *V*_leak_ are the sodium, potassium, and leak reversal potentials, respectively. **Figure** **1**a shows the calculated *g*_Na_, *g*_K_, and *g*_leak_ at steady state (see the Experimental Section). Owing to different reversal potentials, *dV*/*dt* is determined by the contrasting contributions (Figure 1b) of (i) Na^+^ source channel (ρ_+_ > 0) with channel strength *g*_Na_(*V*) = *C*_m_[ρ_+_(*V*)/(*V* − *V*_Na_)] and (ii) K^+^ channel and leak sink channel (ρ_−_ < 0) with channel strengths of *g*_K_(*V*) ≈ −*C*_m_[ρ_−_(*V*)/(*V*_K_ − *V*)] and negligible *g*_leak_ (Note S1 in the Supporting Information for the channel strength). Neuronal operations^1^, ^3^, ^4^, ^5^, ^6^ are thus primarily governed by the competition between Na^+^ and K^+^ channels, which experience “saturation” when *V* →*V*_Na_ and *V* →*V*_K_, respectively (dashed arrows in Figure 1a).

The contrasting and saturable behaviors of ion channels provide guidance for designing neuromorphic wave structures. Because the neuron is an open system interacting with the extracellular region, an open system in non‐Hermitian photonics^18^, ^19^, ^20^ is a suitable platform for realizing photonic neurons. The core of this analogy will then be the construction of state‐dependent “source” and “sink” channels for light. To reproduce the membrane‐potential‐dependent nonlinearity, we utilize light‐intensity‐dependent nonlinearities in photonics, including the Kerr effect,^36^ two‐photon absorption (TPA),^37^ and saturable amplification^23^, ^38^ or absorption.^15^, ^39^, ^40^ Using such nonlinearities, we can assign potential‐dependent “source” and “sink” ion channels to intensity‐dependent “amplifying” and “dissipating” wave channels in the coupled resonator platform (Figure 1c).

A resonator with intensity‐dependent nonlinearities is modeled by the temporal equation,^41^ *d*ψ/*dt* = *iω*_0_ψ + *M*(|ψ|^2^)ψ, where ψ and ω_0_ are the field amplitude and resonant frequency, respectively, and *M*(|ψ|^2^) is the function that determines the type of nonlinearity. The forms of *M*(|ψ|^2^) are classified as (i) a saturable function *M*_Sat_(|ψ|^2^) = γ_Sat_/(1 + |ψ|^2^/|ψ_S_|^2^) with the characteristic intensity |ψ_S_|^2^ or (ii) a Stuart‐Landau (SL) oscillator^5^, ^34^, ^35^, ^42^, ^43^ *M*_S–L_(|ψ|^2^) = γ_S–L_|ψ|^2^, where γ_Sat_ and γ_S–L_ are the strength coefficients. While *M*_S–L_ includes the Kerr effect^36^ with imaginary‐valued γ_S‐L_ and TPA^37^ with real‐valued γ_S‐L_, *M*_Sat_ covers the gain saturation^23^, ^38^ with real‐valued γ_Sat_ > 0 and saturable absorption^15^, ^39^, ^40^ with real‐valued γ_Sat_ < 0.

As the photonic equivalent of the channel strengths *g*_Na_(*V*) and *g*_K_(*V*), we define the “photonic channel strength” as *g*(*I*) = *d*(*dI*/*dt*)/*dI*, where *I* = |ψ|^2^ is the light intensity (see Note S1 in the Supporting Information for comparison). Each type of optical nonlinearity then supports the channel strength *g*_Sat_(*I*) = 2Re[γ_Sat_]/[1 + (*I*/*I*_S_)] with *I*_S_ = |ψ_S_|^2^ and *g*_S–L_(*I*) = 4Re[γ_S–L_]*I*. Considering saturable ion channels, *g*_Sat_(*I*) provides a more suitable fit to *g*_Na_(*V*) and *g*_K_(*V*) than *g*_S–L_(*I*) by assigning gain and loss resonator excitations to *V* → *V*_Na_ and *V* → *V*_K_, respectively (Figure 1d, in comparison with upper and lower illustrations in Figure 1a). To achieve the maximum power transfer between resonators, we set an identical resonant frequency ω_0_ to both resonators,^41^ which leads to the photonic neuron satisfying PT symmetry^18^, ^19^, ^20^, ^21^ with saturable nonlinearities (Figure 1c). The competition between ion channels mediated by Kirchhoff's law is then reproduced by the electromagnetic coupling between resonators.

## 3 Stability Analysis

Saturable gain and loss are quantified by the gain^38^ and loss^44^ coefficients γ_G_,_L_ = γ_G0_,_L0_/(1 + |ψ_G_,_L_/ψ_Gs_,_Ls_|^2^), where γ_G0_,_L0_ are constant coefficients, and |ψ_Gs_,_Ls_|^2^ are saturation intensities. The photonic neuron in Figure 1c is then modeled by the platform‐transparent temporal coupled mode theory (TCMT),^41^ as

(1) $$\frac{d}{dt}\left[\begin{array}{c} \psi_{G}(t)\\ \psi_{L}(t) \end{array}\right]=\left[\begin{array}{cc} i\omega_{0}+\frac{\gamma_{G0}}{1+{\left|\psi_{G}(t)/\psi_{\mathrm{Gs}}\right|}^{2}} & i\kappa \\ i\kappa & i\omega_{0}-\frac{\gamma_{L0}}{1+{\left|\psi_{L}(t)/\psi_{\mathrm{Ls}}\right|}^{2}} \end{array}\right]\;\;\left[\begin{array}{c} \psi_{G}(t)\\ \psi_{L}(t) \end{array}\right]$$

where *ψ*_G_(*t*) and ψ_L_(*t*) are the field amplitudes in gain and loss resonators, respectively, and κ is the evanescent coupling coefficient between them. Equation (1) can be applied to any types of weakly coupled resonant elements in photonics and microwaves, including microcavities,^45^ nanoparticles,^46^ and metamaterials.^47^ We also note that Equation (1) is the generalization of linear two‐level PT‐symmetric systems^27^ to nonlinear domains (ψ_Gs,Ls_ → ∞ for linear systems), covering saturable responses of nonlinear gain and loss coefficients. This extension also allows for the photonic analogy of the competition between saturable ion channels using the coupling coefficient κ between saturable gain and loss elements.

Equation (1) can be divided by separating the amplitude and phase components of light fields^48^ ψ_G,L_ = (*I*_G,L_)^1/2^·exp(*iθ*_G,L_). According to PT‐symmetric phases,^27^ we then derive two real‐valued equations from Equation (1), each for the eigenmodes of “unbroken” and “broken” PT symmetry (see the Experimental Section)

(2) $$\begin{array}{c} \frac{d}{dt}I_{G}=\frac{2\gamma_{G0}}{1+\left(I_{G}/I_{\mathrm{Gs}}\right)}I_{G}-\sqrt{I_{G}I_{L}}\left[\frac{\gamma_{G0}}{1+\left(I_{G}/I_{\mathrm{Gs}}\right)}+\frac{\gamma_{L0}}{1+\left(I_{L}/I_{\mathrm{Ls}}\right)}\right]\\ \frac{d}{dt}I_{L}=-\frac{2\gamma_{L0}}{1+\left(I_{L}/I_{\mathrm{Ls}}\right)}I_{L}+\sqrt{I_{G}I_{L}}\left[\frac{\gamma_{G0}}{1+\left(I_{G}/I_{\mathrm{Gs}}\right)}+\frac{\gamma_{L0}}{1+\left(I_{L}/I_{\mathrm{Ls}}\right)}\right] \end{array}$$

(3) $$\begin{array}{c} \frac{d}{dt}I_{G}=\frac{2\gamma_{G0}}{1+\left(I_{G}/I_{\mathrm{Gs}}\right)}I_{G}-2\kappa \sqrt{I_{G}I_{L}}\\ \frac{d}{dt}I_{L}=-\frac{2\gamma_{L0}}{1+\left(I_{L}/I_{\mathrm{Ls}}\right)}I_{L}+2\kappa \sqrt{I_{G}I_{L}} \end{array}$$

where *I*_Gs,Ls_ = |ψ_Gs,Ls_|^2^. It is noted that the resonant frequency ω_0_ does not affect the dynamics of light intensities inside the photonic neuron.

**Figure 2:**
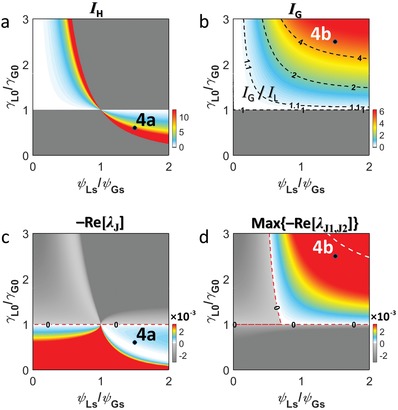
Stability of the equilibria in PT‐symmetric phases. a,b) Equilibrium intensities of a) unbroken (*I*_H_) and b) broken (*I*_G_, *I*_L_) phases (*I*_L_ is not shown). Black dashed contours in (b) denote *I*_G_/*I*_L_. c,d) Jacobian eigenvalues for the stability classification: c) −Re[λ_J_] for the unbroken phase and d) the maximum value of −Re[λ_J1,J2_] for the broken phase. Red dashed lines in (c,d) represent the zero level, and white dashed line in (d) denotes the transition between nodes and foci (Note S2, Supporting Information). Points 4a and 4b in (a–d) denote the cases of Figure 4a,b, respectively. κ = γ_G0_ = 5 × 10^−3^, and ψ_Gs_ = 2 for all cases.

With Equations (2) and (3), we conduct the bifurcation analysis^2^ to examine the stability of PT‐symmetric phases (see the Experimental Section). From Equation (2) with the equilibrium condition *dI*_G,L_/*dt* = 0, we look for the nontrivial equilibrium of the unbroken PT symmetry, which leads to the homogeneous steady state^34^ (HSS) of *I*_G_ = *I*_L_ ≡ *I*_H_: the same light intensity level in gain and loss resonators (Experimental Section). The existence of *I*_H_ is determined by *I*_H_ ≥ 0 (**Figure** **2**a) which automatically satisfies unbroken PT symmetry. On the other hand, Equation (3) with *dI*_G,L_/*dt* = 0 results in a nontrivial equilibrium of the broken PT‐symmetric phase, which leads to *I*_G_ = *I*_L_ at the EP but *I*_G_ ≠ *I*_L_ in the broken PT‐symmetric phase (Experimental Section). In contrast to the HSS in the unbroken phase or at the EP, the broken phase thus supports the inhomogeneous steady state^5^ (IHSS) in the regime of γ_G0_γ_L0_ ≥ κ^2^ for nonnegative *I*_G_ and *I*_L_ (Figure 2b): the different light intensity level in gain and loss resonators.

From the first Lyapunov criterion, we classify the stability of each equilibrium in Figure 2c,d using the Jacobian matrices of Equations (2), (3). First, the Jacobian matrix of the unbroken phase has only one eigenvalue λ_J_ at equilibrium (Experimental Section), forming the hyperbolic equilibrium with γ_G0_ ≠ γ_L0_. Because the stability of hyperbolic equilibria is defined by the sign of the real parts of Jacobian eigenvalues,^2^ the phase transition at the unbroken PT symmetry occurs at γ_G0_ = γ_L0_ (Figure 2c): asymptotically stable with γ_G0_ > γ_L0_ owing to Re[λ_J_] < 0 and unstable with γ_G0_ < γ_L0_. In contrast, equilibrium in the broken phase supports two Jacobian eigenvalues λ_J1_ and λ_J2_ (Experimental Section). Except for the boundary (red dashed lines in Figure 2d), Re[λ_J1,J2_] is nonzero, again corresponding to hyperbolic equilibria. The stability of the equilibria is then classified^2^ as asymptotically stable nodes or foci for Re[λ_J1,J2_] < 0 and unstable foci for Re[λ_J1,J2_] > 0 (Figure 2d, see also Note S2 in the Supporting Information).

**Figure 3:**
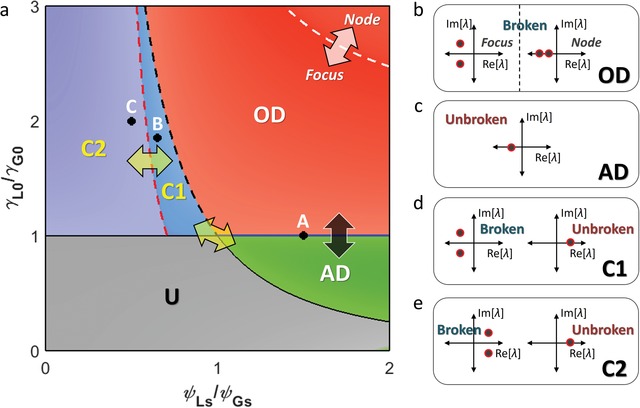
Classification of the dynamics in the photonic neuron. a) Phase diagram defined by the stability of the phase portrait and PT symmetry. Yellow arrows denote the stability transition. The black arrow across the blue solid line represents the transition between AD and OD for both stability and PT‐symmetric transitions. The white arrow across the white dashed line indicates the transition between nodes and foci with the same stability. Red and black dashed lines denote C1–C2 and OD–C1 boundaries, respectively. Black solid lines represent the boundaries around the U phase without nontrivial equilibria. b–d) Illustrations of Jacobian eigenvalues in the complex plane for each phase: b) OD, c) AD, d) C1, and e) C2 phases. Dashed lines in (a) are used when at least one equilibrium preserves its stability condition: from focus to node in (b), from (b) to (d), and from (d) to (e). The dashed line in (b) denotes the node‐focus transition, and the coexistences of unbroken and broken PT‐symmetric equilibria are shown in d) C1 and e) C2 phases.

**Figure** **3**a shows the phase classification of the photonic neuron, achieved from Figure 2. The entire diagram is classified into five phases according to the stability of equilibria and PT symmetry: (i) the OD phase with stable IHSS of broken PT symmetry (Figure 3b), (ii) the AD phase with stable HSS of unbroken PT symmetry (Figure 3c), (iii) the C1 (Figure 3d) and (iv) C2 (Figure 3e) phases with the coexisting nontrivial equilibria of unbroken and broken PT‐symmetric phases, and (v) the U unstable phase without any nontrivial equilibrium. Each phase boundary originates from a different physical origin with respect to stability and PT symmetry. Phase transitions for the given PT‐symmetric phase (yellow arrows) occur at the interfaces between the AD and C1–C2 phases (unbroken) and between the OD–C1 and C2 phases (broken). The interface between the AD and OD phases (black arrow) leads to both stability and PT‐symmetric phase transitions across the EP. While the OD phase can be divided into two subclasses for stable nodes and foci (white arrow), these subclasses exhibit the same stability condition (Note S2, Supporting Information).

## 4 Photonic Oscillation Quenching

**Figure 4:**
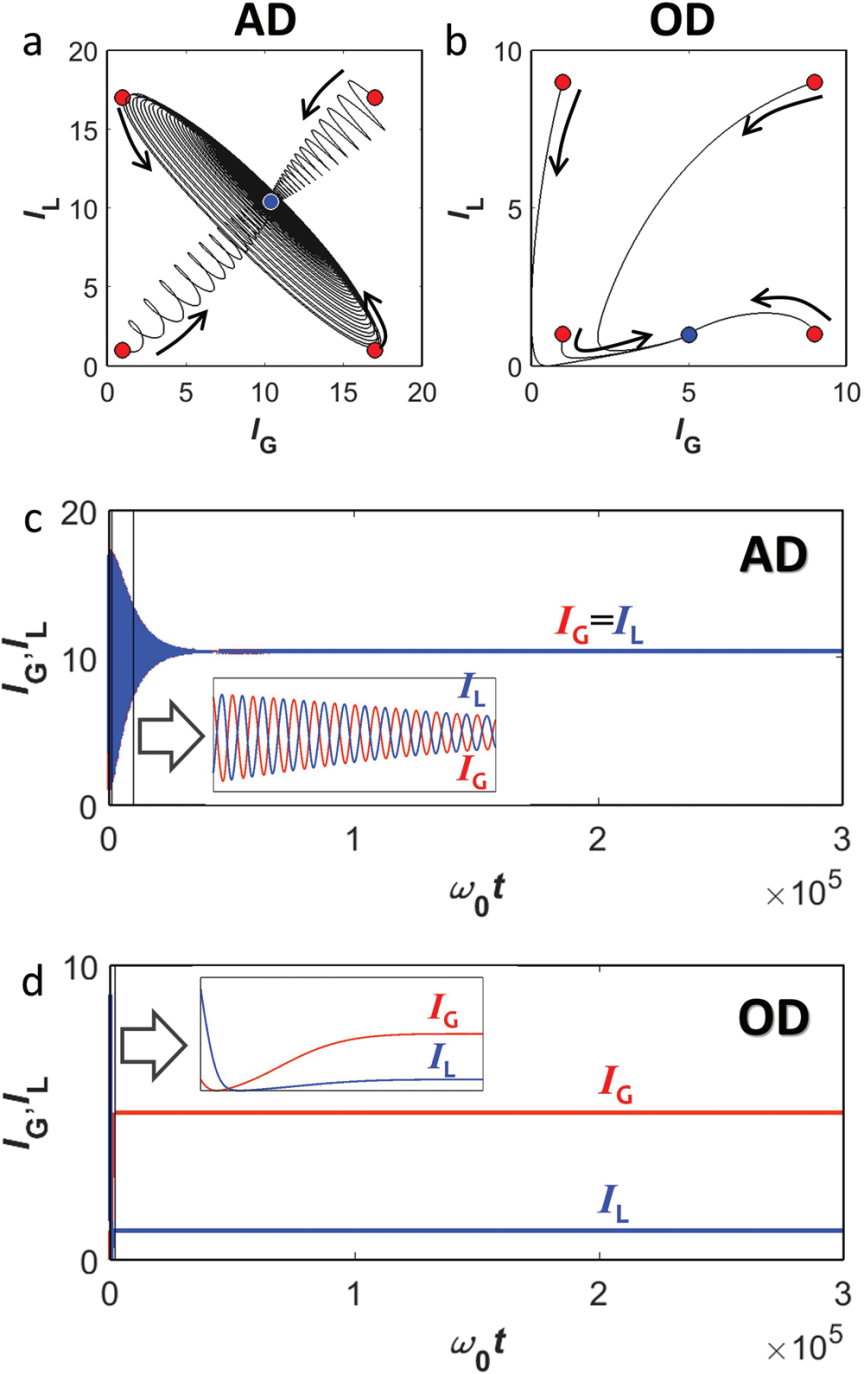
Oscillation quenching phenomena in the photonic neuron. Phase portraits on the *I*_G_–*I*_L_ space: a) AD phase in unbroken PT symmetry and b) OD phase in broken PT symmetry, each for the points 4a and 4b in Figure 2a–d. Red (or blue) circles represent the initial (or final) states, and black arrows depict the direction of evolution. c,d) Examples of calculated temporal dynamics of c) AD and d) OD. The results in (a–d) are obtained by the time domain simulation of Equation (1) using the 6th order Runge–Kutta method^49^ with the unit step 2π/(200ω_0_). Black lines in (a,b) denote the loci during 10^7^ time steps. The initial condition in (c,d) is [*I*_G_, *I*_L_] = [1,17] for (c) and [*I*_G_, *I*_L_] = [1,9] for (d). The enlarged plots represent the temporal dynamics during 10^3^ ≤ ω_0_ *t* ≤ 10^4^ for (c) and 0 ≤ ω_0_ *t* ≤ 2 × 10^3^ for (d). κ = γ_G0_ = 5 × 10^−3^, and ψ_Gs_ = 2 for all cases. The initial phase difference between resonators is assigned according to the procedure in the Experimental Section.

In the phase classification of Figure 3, we examine wave behaviors in AD and OD phases, each representing distinct oscillation quenching phenomena. In the regime of γ_G0_ > γ_L0_ with the stable HSS *I*_H_ (e.g., point 4a in Figure 2a,c), the calculated phase portrait shows the robust convergence of *I*_G_ and *I*_L_ to the same value of *I*_H_ (**Figure** **4**a), which derives AD oscillation quenching^34^ in unbroken PT symmetry: the identical constant excitation of resonators with *I*_H_. In contrast, in the regime of γ_G0_ < γ_L0_ with the stable IHSS *I*_G_ ≠ *I*_L_ (e.g., point 4b in Figure 2b,d), numerical analysis proves the robust convergences to the equilibrium of *I*_G_ ≠ *I*_L_ independent from initial *I*_G_ and *I*_L_ (Figure 4b), which allows OD oscillation quenching^35^ in broken PT symmetry: the constant excitation of resonators with different intensity states *I*_G_ ≠ *I*_L_.

We demonstrate these oscillation quenching functions in Figure 4c,d with the time domain simulation, showing photonic AD and OD, respectively. The results in Figure 2a–d provide the design criteria of AD and OD phenomena in the photonic neuron in terms of the intensity level (Figure 2a,b) and the types of the phase portrait defined by Jacobian eigenvalues (Figure 2c,d). It is also noted that the AD‐OD transition boundary (black arrow in Figure 3a) at the EP represents the complete suppression of light fields inside resonators (Note S3, Supporting Information for point A in Figure 3a) due to the transition from the nontrivial to trivial equilibrium [*I*_G_, *I*_L_] = [0, 0] for the continuous transition between AD (*I*_G_ = *I*_L_) and OD (*I*_G_ ≠ *I*_L_).

## 5 Photonic Repetitive Firing at the Coexisting Phase

In contrast to the AD and OD phases, which support asymptotically stable equilibria from either unbroken or broken PT symmetry, the C1 and C2 phases defined by the coexistence of the equilibria from both unbroken and broken PT symmetries cannot be described solely by Equations (2) or (3). Phase portraits in these coexisting phases are strongly dependent on the initial state of light. First, consider the C1 phase defined by the stable equilibrium of the broken PT‐symmetric phase and the unstable equilibrium of the unbroken phase. The PT‐symmetric phase between being stable broken and unstable unbroken is dependent on the initial condition (Note S4 in the Supporting Information for point B in Figure 3a), allowing the turning “on” and “off” behavior of the OD oscillation quenching functions.

We now focus on the coexisting phase C2, offering nonreciprocal oscillations which are absent from other dynamic phases (AD, OD, C1, and U phases). This reveals the critical role of the saturable absorption (finite value of ψ_Ls_) for nonreciprocal oscillations, because the gain saturation only (finite ψ_Gs_ and ψ_Ls_ → ∞) cannot lead to the C2 phase. In contrast to the C1 phase, the C2 phase possesses unstable equilibria of both the unbroken and broken PT‐symmetric phases, and this instability allows the dynamic transition between PT‐symmetric phases.

**Figure 5:**
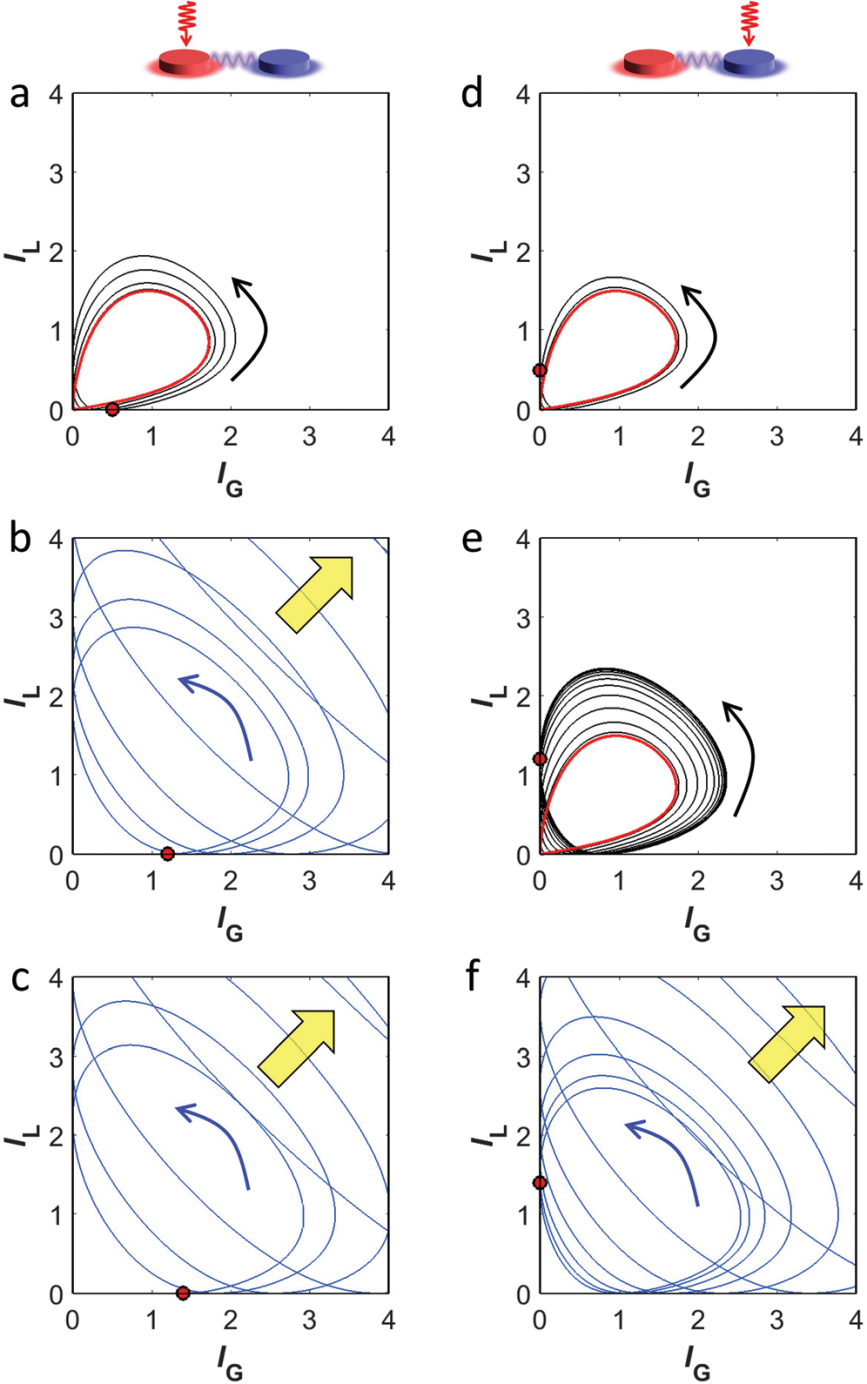
Limit cycle phase portraits in the *I*_G_–*I*_L_ space: a–c) gain and d–f) loss resonator excitation for point C in Figure 3a, obtained by solving Equation (1). The initial light intensities are a,d) 0.5, b,e) 1.2, and c,f) 1.4. The black (or red) lines in (a,d,e) denote the convergent loci (or limit cycle) of the intensity state, and the blue lines in (b,c,f) denote the divergent loci during 10^7^ time steps with the unit step 2π/(200ω_0_). The black and blue arrows depict the direction of evolution, and the yellow arrows represent the divergence of intensities. The red circles represent the initial intensity state.

**Figure** **5** shows the phase portraits of the C2 phase with gain (Figure 5a–c) and loss (Figure 5d–f) resonator excitations. The limit cycle solutions are achieved at low intensity (red lines in Figure 5a,d), while the system diverges at high intensity (Figure 5c,f). The limit cycle that allows the repetitive firing of light is a unique feature of the C2 phase originating from the “oscillatory” PT‐symmetric phase transition across the EP (Note S5, Supporting Information). The transition between the limit cycle and instability occurs at different intensity levels of the excitation port (Figure 5b,e), which imposes directionality on the photonic neuron. We also note that the convergence “speed” to the limit cycle in the repetitive firing is manipulated dependent on the initial intensity (Figure 5d,e). Therefore, with identical limit cycles that have different convergence times, we can develop the tuning of the temporal phase of photonic repetitive firing with the full coverage (Notes S6 and S7 in the Supporting Information).

## 6 Conclusion

In summary, we investigated the dynamic functions in photonic neurons with saturable gain and loss channels. We developed the phase diagram of photonic neuronal dynamics in terms of its phase portrait. Each phase exhibits different stability and equilibrium conditions in relation to the PT‐symmetric phase. The connection between the PT‐symmetric phase transition and AD‐OD transition was revealed, providing the design criteria of photonic oscillation quenching functions. In the coexisting phase, we also demonstrated the repetitive firing of light with directionality and tunable time delay.

As shown in the platform‐transparent TCMT equation and general stability theory, our analysis can be applied to various optical elements in the weak coupling regime. This enables the utilization of well‐established saturable media and structures, such as pumped media,^23^, ^38^ organic dyes,^39^ graphene layers,^15^ and artificial realizations^40^ for the construction of nonlinear PT‐symmetric systems for neuromorphic photonics.

Our proposal of PT‐symmetric dynamics will inspire new approaches for the design of a photonic neuron or its network, as demonstrated in the robust stability nature of photonic oscillation quenching and strong nonreciprocal repetitive firing. It is expected that neuronal functionalities of electromagnetic waves could be used as building blocks for neuromorphic wave circuits, underpinning the critical role for oscillation quenching, network synchronizations,^50^ and weighted and directional graph networks.^51^

## 7 Experimental Section

*Nonlinear Conductance of the Neuron*: According to the HH model,^1^ the Na^+^ and K^+^ ion channel strengths are given by *g*_Na_(*V*) = *g*_Na_^0^·*m* ^3^ *h* and *g*_K_(*V*) = *g*_K_^0^·*n* ^4^, respectively, where

(4) $$\frac{\partial X}{\partial t}=\alpha_{X}\left(V\right)\left(1-X\right)-\beta_{X}\left(V\right)X$$

with *X* = *m*, *n*, and *h*, and

(5) $$\begin{array}{l} \alpha_{m}=\frac{0.1\left(25-V\right)}{\exp\left[\left(25-V\right)/10\right]-1},\;\;\;\;\;\;\beta_{m}=4\exp\left(-V/18\right)\\ \alpha_{n}=\frac{0.01\left(10-V\right)}{\exp\left[\left(10-V\right)/10\right]-1},\;\;\;\;\;\;\;\beta_{n}=0.125\exp\left(-V/80\right)\\ \alpha_{h}=0.07\exp\left(-V/20\right),\;\;\;\;\;\;\;\;\;\;\;\;\beta_{h}=\frac{1}{\exp\left[\left(30-V\right)/10\right]+1} \end{array}$$

The steady‐state condition (*∂*/*∂t* → 0) leads to *X*(*V*) = *α_X_*(*V*)/[*α_X_*(*V*) + *β_X_*(*V*)], clarifying the state dependency of the ion channel strengths. To calculate the result in Figure 1a, the parameters used were: *g*_Na_^0^ = 120 mS·cm^−2^, *g*_K_^0^ = 36 mS·cm^−2^, and *g*_leak_ = 0.3 mS·cm^−2^, *V*_Na_ = 115 mV, *V*_K_ = −12 mV, *V*_Na_ = 10.599 mV, and *C*_m_ = 1 µF·cm^−2^.

*Dynamic Intensity Equations for PT‐Symmetric Phases*: From Equation (1) and the relation of *d*|ψ|^2^/*dt* = 2*Re*[ψ*dψ/*dt*], the following real‐valued intensity equation was derived

(6) $$\frac{d}{dt}\left[\begin{array}{c} I_{G}(t)\\ I_{L}(t) \end{array}\right]=\left[\begin{array}{cc} \frac{2\gamma_{G0}I_{\mathrm{Gs}}}{I_{\mathrm{Gs}}+I_{G}(t)} & 2\kappa \sqrt{I_{G}(t)I_{L}(t)}\sin\left(\theta_{G}-\theta_{L}\right)\\ -2\kappa \sqrt{I_{G}(t)I_{L}(t)}\sin\left(\theta_{G}-\theta_{L}\right) & -\frac{2\gamma_{L0}I_{\mathrm{Ls}}}{I_{\mathrm{Ls}}+I_{L}(t)} \end{array}\right]\;\;\left[\begin{array}{c} I_{G}(t)\\ I_{L}(t) \end{array}\right]$$

where θ_G_ and θ_L_ are the real‐valued phase of the field amplitude inside each optical resonator, as ψ_G,L_ = (*I*_G,L_)^1/2^·exp(*iθ*_G,L_), resulting in the relation of the local phase difference θ_G_ − θ_L_ = Im[log(ψ_G_/ψ_L_)]. Equation (6) can be simplified for the eigenmodes of the system, which are obtained from the harmonic approximation of Equation (1). To achieve this, the instantaneous eigenfrequencies were derived: ω_e_(*t*) = ω_0_ − *iκγ*_avg_(*t*) ± κ·[*D*(*t*)]^1/2^ of the system at time *t*, from *d*ψ/*dt* → *iω*_e_ψ, where

(7) $$\gamma_{\mathrm{avg}}\left(t\right)=\frac{\frac{\gamma_{G0}I_{\mathrm{Gs}}}{I_{\mathrm{Gs}}+I_{G}\left(t\right)}-\frac{\gamma_{L0}I_{\mathrm{Ls}}}{I_{\mathrm{Ls}}+I_{L}\left(t\right)}}{2\kappa },\;D\left(t\right)=1-{\left(\frac{\frac{\gamma_{G0}I_{\mathrm{Gs}}}{I_{\mathrm{Gs}}+I_{G}\left(t\right)}+\frac{\gamma_{L0}I_{\mathrm{Ls}}}{I_{\mathrm{Ls}}+I_{L}\left(t\right)}}{2\kappa }\right)}^{2}$$

The time‐varying function γ_avg_(*t*) determines the instantaneous gauge of the PT‐symmetric system, as γ_avg_ > 0 for the active regime, γ_avg_ < 0 for the passive regime, and γ_avg_ = 0 for the normal PT symmetry. On the other hand, *D*(*t*) defines the phase of PT symmetry:^27^ *D*(*t*) > 0 for the unbroken phase, *D*(*t*) < 0 for the broken phase, and *D*(*t*) = 0 for the PT‐symmetric phase transition at the EP. Notably, the local phase difference θ_G_ − θ_L_ of the eigenmode is uniquely defined by the phase of PT symmetry.^27^ Using the instantaneous eigenmodes of the system obtained from Equation (1) for both eigenfrequencies ω_e_(*t*)

(8) $$\sin\left(\theta_{G}-\theta_{L}\right)=-\frac{\frac{\gamma_{G0}I_{\mathrm{Gs}}}{I_{\mathrm{Gs}}+I_{G}\left(t\right)}+\frac{\gamma_{L0}I_{\mathrm{Ls}}}{I_{\mathrm{Ls}}+I_{L}\left(t\right)}}{2\kappa }$$

(9) $$\sin\left(\theta_{G}-\theta_{L}\right)=-1$$

each for the unbroken and broken PT‐symmetric phases, deriving Equations (2) and (3) in the main text from Equation (6), respectively.

*Nontrivial Equilibrium in PT‐Symmetric Phases*: From *dI*_G,L_/*dt* = 0 in Equations (2), (3) for the condition of the equilibrium, the following relations are obtained for unbroken and broken phases, respectively

(10) $$\frac{2\gamma_{G0}I_{\mathrm{Gs}}}{I_{\mathrm{Gs}}+I_{G}}I_{G}=\frac{2\gamma_{L0}I_{\mathrm{Ls}}}{I_{\mathrm{Ls}}+I_{L}}I_{L}=\sqrt{I_{G}I_{L}}\left(\frac{\gamma_{G0}I_{\mathrm{Gs}}}{I_{\mathrm{Gs}}+I_{G}}+\frac{\gamma_{L0}I_{\mathrm{Ls}}}{I_{\mathrm{Ls}}+I_{L}}\right)$$

(11) $$\frac{2\gamma_{G0}I_{\mathrm{Gs}}}{I_{\mathrm{Gs}}+I_{G}}I_{G}=\frac{2\gamma_{L0}I_{\mathrm{Ls}}}{I_{\mathrm{Ls}}+I_{L}}I_{L}=2\kappa \sqrt{I_{G}I_{L}}$$

Equation (10) leads to *I*_G_ = *I*_L_ ≡ *I*_H_ = (γ_G0_ − γ_L0_)*I*_Gs_ *I*_Ls_/(γ_L0_ *I*_Ls_ − γ_G0_ *I*_Gs_) for unbroken PT symmetry, while Equation (11) derives the equilibrium for broken PT symmetry, as

(12) $$\begin{array}{l} I_{G}=\frac{1}{2}\frac{\gamma_{G0}}{\kappa }\frac{I_{\mathrm{Gs}}}{I_{\mathrm{Ls}}}\left[\sqrt{{\left(\frac{\gamma_{G0}I_{\mathrm{Gs}}-\gamma_{L0}I_{\mathrm{Ls}}}{\kappa }\right)}^{2}+4I_{\mathrm{Gs}}I_{\mathrm{Ls}}}-\frac{\gamma_{G0}I_{\mathrm{Gs}}-\gamma_{L0}I_{\mathrm{Ls}}}{\kappa }-2\frac{\kappa }{\gamma_{G0}}I_{\mathrm{Ls}}\right]\\ I_{L}=\frac{1}{2}\frac{\gamma_{L0}}{\kappa }\frac{I_{\mathrm{Ls}}}{I_{\mathrm{Gs}}}\left[\sqrt{{\left(\frac{\gamma_{G0}I_{\mathrm{Gs}}-\gamma_{L0}I_{\mathrm{Ls}}}{\kappa }\right)}^{2}+4I_{\mathrm{Gs}}I_{\mathrm{Ls}}}+\frac{\gamma_{G0}I_{\mathrm{Gs}}-\gamma_{L0}I_{\mathrm{Ls}}}{\kappa }-2\frac{\kappa }{\gamma_{L0}}I_{\mathrm{Gs}}\right] \end{array}$$

It is noted that the condition of broken PT symmetry except the EP, κ < *F*(*I*_G_, *I*_L_), where *F*(*I*_G_, *I*_L_) = [γ_G0_ *I*_Gs_/(*I*_Gs_ + *I*_G_) + γ_L0_ *I*_Ls_/(*I*_Ls_ + *I*_L_)]/2, enforces the IHSS *I*_G_ ≠ *I*_L_ from Equations (11) and (12).

*Stability Analysis from the First Lyapunov Criterion*: To examine the stability of the obtained equilibria, the Jacobian matrix is derived^2^ as the linearization of the PT‐symmetric system. From Equation (2) of the unbroken PT‐symmetric phase, the Jacobian matrix *A* at equilibrium *I*_G_ = *I*_L_ = *I*_H_ = (γ_G0_ − γ_L0_)*I*_Gs_ *I*_Ls_/(γ_L0_ *I*_Ls_ − γ_G0_ *I*_Gs_) becomes

(13) $$A=\left[\begin{array}{c} \frac{M^{2}}{\gamma_{\mathrm{G0}}}\\ \frac{M^{2}}{\gamma_{\mathrm{G0}}} \end{array}\begin{array}{c} -\frac{M^{2}}{\gamma_{\mathrm{L0}}}\\ -\frac{M^{2}}{\gamma_{\mathrm{L0}}} \end{array}\right]$$

where *M* = γ_G0_ *I*_Gs_/(*I*_Gs_ + *I*_H_) = γ_L0_ *I*_Ls_/(*I*_Ls_ + *I*_H_). Therefore, the Jacobian matrix of the unbroken PT‐symmetric phase becomes 1‐dimensional due to the linear dependence, and its unique eigenvalue λ_J_ becomes

(14) $$\lambda_{J}=\left(\gamma_{\mathrm{L0}}-\gamma_{\mathrm{G0}}\right)\frac{I_{\mathrm{Gs}}I_{\mathrm{Ls}}}{\left(I_{\mathrm{Gs}}+I_{H}\right)\left(I_{\mathrm{Ls}}+I_{H}\right)}$$

Due to the first Lyapunov criterion,^2^ the PT‐symmetric neuron at the unbroken phase becomes asymptotically stable with Re[λ_J_] < 0, which is satisfied by γ_L0_ > γ_G0_, *I*_Gs_ ≠ 0 and *I*_Ls_ ≠ 0. In contrast, Equation (3) derives the Jacobian matrix *A* for the broken PT‐symmetric phase at equilibrium, as

(15) $$A=\left[\begin{array}{c} \frac{2\gamma_{\mathrm{G0}}I_{\mathrm{Gs}}{}^{2}}{{\left(I_{\mathrm{Gs}}+I_{G}\right)}^{2}}-\kappa \sqrt{\frac{I_{L}}{I_{G}}}\\ \kappa \sqrt{\frac{I_{L}}{I_{G}}} \end{array}\begin{array}{c} -\kappa \sqrt{\frac{I_{G}}{I_{L}}}\\ -\frac{2\gamma_{\mathrm{L0}}I_{\mathrm{Ls}}{}^{2}}{{\left(I_{\mathrm{Ls}}+I_{L}\right)}^{2}}+\kappa \sqrt{\frac{I_{G}}{I_{L}}} \end{array}\right]$$

where *I*_G_ and *I*_L_ at equilibrium are defined by Equation (12). The matrix *A* of Equation (15) then supports two eigenvalues, as

(16) $$\begin{array}{l} \lambda_{\mathrm{J1},\mathrm{J2}}=\frac{1}{2}\{\kappa \left(\sqrt{\frac{I_{G}}{I_{L}}}-\sqrt{\frac{I_{L}}{I_{G}}}\right)+\left[\frac{2\gamma_{\mathrm{G0}}I_{\mathrm{Gs}}{}^{2}}{{\left(I_{\mathrm{Gs}}+I_{G}\right)}^{2}}-\frac{2\gamma_{\mathrm{L0}}I_{\mathrm{Ls}}{}^{2}}{{\left(I_{\mathrm{Ls}}+I_{L}\right)}^{2}}\right]\\ \pm \sqrt{\kappa \left(\sqrt{\frac{I_{G}}{I_{L}}}+\sqrt{\frac{I_{L}}{I_{G}}}\right)-\left[\frac{2\gamma_{\mathrm{G0}}I_{\mathrm{Gs}}{}^{2}}{{\left(I_{\mathrm{Gs}}+I_{G}\right)}^{2}}+\frac{2\gamma_{\mathrm{L0}}I_{\mathrm{Ls}}{}^{2}}{{\left(I_{\mathrm{Ls}}+I_{L}\right)}^{2}}\right]-4\kappa^{2}}\} \end{array}$$

The PT‐symmetric neuron at the broken phase then becomes asymptotically stable with Re[λ_J1_] < 0 and Re[λ_J2_] < 0.

## Conflict of Interest

The authors declare no conflict of interest.

## Supporting information

Supplementary

## References

[1] A. L. Hodgkin , A. F. Huxley , J. Physiol. 1952, 117, 500. 12991237 10.1113/jphysiol.1952.sp004764 PMC1392413

[2] Y. A. Kuznetsov , Elements of Applied Bifurcation Theory, Vol. 112, Springer Science & Business Media, Berlin, Germany 2013.

[3] J. Wang , L. Chen , X. Fei , Chaos, Solitons Fractals 2007, 33, 217.

[4] R. Guttman , S. Lewis , J. Rinzel , J. Physiol. 1980, 305, 377. 7441560 10.1113/jphysiol.1980.sp013370 PMC1282979

[5] A. Koseska , E. Volkov , J. Kurths , Phys. Rep. 2013, 531, 173.

[6] E. Rossoni , Y. Chen , M. Ding , J. Feng , Phys. Rev. E 2005, 71, 061904. 10.1103/PhysRevE.71.061904 16089762

[7] M. Mahowald , R. Douglas , Nature 1991, 354, 515. 1661852 10.1038/354515a0

[8] J. Torrejon , M. Riou , F. A. Araujo , S. Tsunegi , G. Khalsa , D. Querlioz , P. Bortolotti , V. Cros , K. Yakushiji , A. Fukushima , Nature 2017, 547, 428. 28748930 10.1038/nature23011 PMC5575904

[9] P. R. Prucnal , B. J. Shastri , Neuromorphic Photonics, CRC Press, Boca Raton, FL, USA 2017.

[10] P. R. Prucnal , B. J. Shastri , T. F. de Lima , M. A. Nahmias , A. N. Tait , Adv. Opt. Photonics 2016, 8, 228.

[11] A. N. Tait , M. A. Nahmias , Y. Tian , B. J. Shastri , P. R. Prucnal , in Nanophotonic Information Physics (Eds: M. Naruse), Springer, Berlin, Germany 2014, Ch. 8.

[12] A. Yariv , P. Yeh , Photonics: Optical Electronics in Modern Communications, Vol. 6, Oxford University Press, New York 2007.

[13] H. A. Haus , J. Appl. Phys. 1975, 46, 3049.

[14] A. Pusch , S. Wuestner , J. M. Hamm , K. L. Tsakmakidis , O. Hess , ACS Nano 2012, 6, 2420. 22329714 10.1021/nn204692x

[15] Q. Bao , H. Zhang , Y. Wang , Z. Ni , Y. Yan , Z. X. Shen , K. P. Loh , D. Y. Tang , Adv. Funct. Mater. 2009, 19, 3077.

[16] M. Zhang , Q. Wu , F. Zhang , L. Chen , X. Jin , Y. Hu , Z. Zheng , H. Zhang , Adv. Opt. Mater. 2019, 7, 1800224.

[17] Y. Shen , N. C. Harris , S. Skirlo , M. Prabhu , T. Baehr‐Jones , M. Hochberg , X. Sun , S. Zhao , H. Larochelle , D. Englund , M. Soljačić , Nat. Photonics 2017, 11, 441.

[18] L. Feng , R. El‐Ganainy , L. Ge , Nat. Photonics 2017, 11, 752.

[19] R. El‐Ganainy , K. G. Makris , M. Khajavikhan , Z. H. Musslimani , S. Rotter , D. N. Christodoulides , Nat. Phys. 2018, 14, 11.

[20] Ş. K. Özdemir , S. Rotter , F. Nori , L. Yang , Nat. Mater. 2019, 10.1038/s41563-019-0304-9. 30962555

[21] C. M. Bender , S. Boettcher , Phys. Rev. Lett. 1998, 80, 5243.

[22] B. Peng , Ş. K. Özdemir , F. Lei , F. Monifi , M. Gianfreda , G. L. Long , S. Fan , F. Nori , C. M. Bender , L. Yang , Nat. Phys. 2014, 10, 394.

[23] L. Chang , X. Jiang , S. Hua , C. Yang , J. Wen , L. Jiang , G. Li , G. Wang , M. Xiao , Nat. Photonics 2014, 8, 524.

[24] A. U. Hassan , B. Zhen , M. Soljačić , M. Khajavikhan , D. N. Christodoulides , Phys. Rev. Lett. 2017, 118, 093002. 28306295 10.1103/PhysRevLett.118.093002

[25] S. Assawaworrarit , X. Yu , S. Fan , Nature 2017, 546, 387. 28617463 10.1038/nature22404

[26] M.‐A. Miri , A. Alù , Science 2019, 363, eaar7709. 30606818 10.1126/science.aar7709

[27] C. E. Rüter , K. G. Makris , R. El‐Ganainy , D. N. Christodoulides , M. Segev , D. Kip , Nat. Phys. 2010, 6, 192.

[28] W. Chen , Ş. K. Özdemir , G. Zhao , J. Wiersig , L. Yang , Nature 2017, 548, 192. 28796206 10.1038/nature23281

[29] A. Regensburger , C. Bersch , M. A. Miri , G. Onishchukov , D. N. Christodoulides , U. Peschel , Nature 2012, 488, 167. 22874962 10.1038/nature11298

[30] M. Lawrence , N. Xu , X. Zhang , L. Cong , J. Han , W. Zhang , S. Zhang , Phys. Rev. Lett. 2014, 113, 093901. 25215984 10.1103/PhysRevLett.113.093901

[31] S. Yu , H. S. Park , X. Piao , B. Min , N. Park , Optica 2016, 3, 1025.

[32] H. Hodaei , M.‐A. Miri , M. Heinrich , D. N. Christodoulides , M. Khajavikhan , Science 2014, 346, 975. 25414308 10.1126/science.1258480

[33] J. Doppler , A. A. Mailybaev , J. Böhm , U. Kuhl , A. Girschik , F. Libisch , T. J. Milburn , P. Rabl , N. Moiseyev , S. Rotter , Nature 2016, 537, 76. 27454554 10.1038/nature18605

[34] G. Saxena , A. Prasad , R. Ramaswamy , Phys. Rep. 2012, 521, 205.

[35] A. Koseska , E. Volkov , J. Kurths , Phys. Rev. Lett. 2013, 111, 024103. 23889406 10.1103/PhysRevLett.111.024103

[36] H. Kang , Y. Zhu , Phys. Rev. Lett. 2003, 91, 093601. 14525180 10.1103/PhysRevLett.91.093601

[37] H. K. Tsang , C. Wong , T. Liang , I. Day , S. Roberts , A. Harpin , J. Drake , M. Asghari , Appl. Phys. Lett. 2002, 80, 416.

[38] L. Yang , T. Carmon , B. Min , S. M. Spillane , K. J. Vahala , Appl. Phys. Lett. 2005, 86, 091114.

[39] E. Spiller , J. Appl. Phys. 1972, 43, 1673.

[40] M. Teimourpour , A. Rahman , K. Srinivasan , R. El‐Ganainy , Phys. Rev. Appl. 2017, 7, 014015. 28580374 10.1103/PhysRevApplied.7.014015 PMC5455789

[41] H. A. Haus , Waves and Fields in Optoelectronics, Vol. 464, Prentice‐Hall, Englewood Cliffs, NJ 1984.

[42] C. R. Mirasso , P. V. Carelli , T. Pereira , F. S. Matias , M. Copelli , Chaos: Interdiscip. J. Nonlinear Sci. 2017, 27, 114305. 10.1063/1.5006932 29195321

[43] A. Röhm , K. Lüdge , I. Schneider , Chaos: Interdiscip. J. Nonlinear Sci. 2018, 28, 063114. 10.1063/1.5018262 29960415

[44] M. Hercher , Appl. Opt. 1967, 6, 947. 20057881 10.1364/AO.6.000947

[45] B. Maes , M. Soljacic , J. D. Joannopoulos , P. Bienstman , R. Baets , S.‐P. Gorza , M. Haelterman , Opt. Express 2006, 14, 10678. 19529474 10.1364/oe.14.010678

[46] P. T. Bowen , D. R. Smith , Phys. Rev. B 2014, 90, 195402.

[47] A. A. Sukhorukov , A. S. Solntsev , S. S. Kruk , D. N. Neshev , Y. S. Kivshar , Opt. Lett. 2014, 39, 462. 24487840 10.1364/OL.39.000462

[48] S. Yu , X. Piao , N. Park , Phys. Rev. Lett. 2018, 120, 193902. 29799257 10.1103/PhysRevLett.120.193902

[49] J. C. Butcher , J. Aust. Math. Soc. 1964, 4, 179.

[50] W. Wang , G. Chen , Z. Wang , Phys. Rev. E 1997, 56, 3728.

[51] T. L. Fine , Feedforward Neural Network Methodology, Springer Science & Business Media, Berlin, Germany 2006.

