# Supplementary material for: Neuromorphic Functions of Light in Parity‐Time‐Symmetric Systems
Source: Adv Sci (Weinh). 2019 Jun 3;6(15):1900771. doi: 10.1002/advs.201900771 (PMC6685464; doi:10.1002/advs.201900771)
Supplement: Supplementary file 1 — Supplementary [file ADVS-6-1900771-s001.pdf]

Copyright WILEY-VCH Verlag GmbH & Co. KGaA, 69469 Weinheim, Germany, 2019.

ADVANCED SCIENCE
Open Access

Supporting Information

for *Adv. Sci.,* DOI: 10.1002/advs.201900771

Neuromorphic Functions of Light in Parity-Time-Symmetric Systems

*Sunkyu Yu, Xianji Piao, and Namkyoo Park**

Copyright WILEY-VCH Verlag GmbH & Co. KGaA, 69469 Weinheim, Germany, 2019.

Supporting Information

Neuromorphic Functions of Light in Parity-Time-Symmetric Systems

*Sunkyu Yu, Xianji Piao, and Namkyoo Park**

Photonic Systems Laboratory, Department of Electrical and Computer Engineering, Seoul National University, Seoul 08826, Korea
E-mail: nkpark@snu.ac.kr

Note S1. Ion channel strengths of the neuron

Note S2. Stability of the broken PT-symmetric phase

Note S3. AD-OD phase transition boundary

Note S4. C1 coexisting phase

Note S5. Oscillatory PT-symmetric phase transition

Note S6. Tunability and directionality in photonic repetitive firing

Note S7. Temporal phase differences in field and amplitude

Note S1. Ion channel strengths of the neuron

In the temporal variation of the membrane potential $dV/dt = \rho_+(V) + \rho_-(V)$, we define the ion channel strength as $d(dV/dt)/dV$, which measures the "state-dependent change" in current flow. To handle the source and sink channels separately, we introduce the transformed potentials $V' = V_{\mathrm{Na}} - V$ and $V'' = V - V_{\mathrm{K}}$, each defining the source and sink channel strength as $d\rho_+/dV'$ and $d\rho_-/dV''$. From the relations of $\rho_+(V) = g_{\mathrm{Na}}(V)\cdot(V_{\mathrm{Na}} - V)/C_{\mathrm{m}}$ and $\rho_-(V) = -[g_{\mathrm{K}}(V)\cdot(V - V_{\mathrm{K}}) + g_{\mathrm{leak}}(V - V_{\mathrm{leak}})]/C_{\mathrm{m}}$ shown in the main text, we obtain

$$\begin{aligned} \frac{d\rho_+}{dV'} &= \frac{g_{\mathrm{Na}}}{C_m} + \frac{(V_{\mathrm{Na}} - V)}{C_m}\frac{dg_{\mathrm{Na}}}{dV} \approx \frac{g_{\mathrm{Na}}}{C_m} \\ \frac{d\rho_-}{dV''} &= -\frac{g_{\mathrm{K}}}{C_m} - \frac{(V - V_{\mathrm{K}})}{C_m}\frac{dg_{\mathrm{K}}}{dV} - \frac{g_{\mathrm{leak}}}{C_m} \approx -\frac{g_{\mathrm{K}}}{C_m} \end{aligned} \tag{S1}$$

due to sufficiently slow variations of g_{Na} and g_{K} with respect to V and negligible g_{leak}. Therefore, the nonlinear conductance of $g_{\mathrm{Na}}(V)$ and $g_{\mathrm{K}}(V)$, respectively, operate as the source and sink channel strengths of the neuron.

Note S2. Stability of the broken PT-symmetric phase

In sharp contrast to the unbroken PT-symmetric phase, which possesses a single Jacobian eigenvalue λ_J, the broken PT-symmetric phase with two Jacobian eigenvalues $\lambda_{J1,J2}$ requires a more complicated classification of the stability of the phase portrait in the light intensity space [I_G, I_L]. Figure S1a-d shows the real and imaginary parts of the Jacobian eigenvalues $\lambda_{J1,J2}$ in the broken phase. In the regime of $\gamma_{G0}\gamma_{L0} \geq \kappa^2$ for nonnegative I_G and I_L ($\gamma_{L0} / \gamma_{G0} \geq 1$ in Figure S1 for $\kappa = \gamma_{G0} = 5\times10^{-3}$), there exists the boundary of Re[$\lambda_{J1,J2}$] = 0 in Figure S1a,b, which divides the broken phase into the regimes of stable (chromatic colored regions) and unstable (grey regions) equilibria. According to the imaginary parts (Figure S1c,d) and the differences in each component (Figure S1e,f), the stable regime is again divided into (i) the complex conjugate relation $\lambda_{J1} = \lambda_{J2}^*$ and (ii) Im[$\lambda_{J1,J2}$] = 0 with different real parts Re[λ_{J1}] ≠ Re[λ_{J2}].

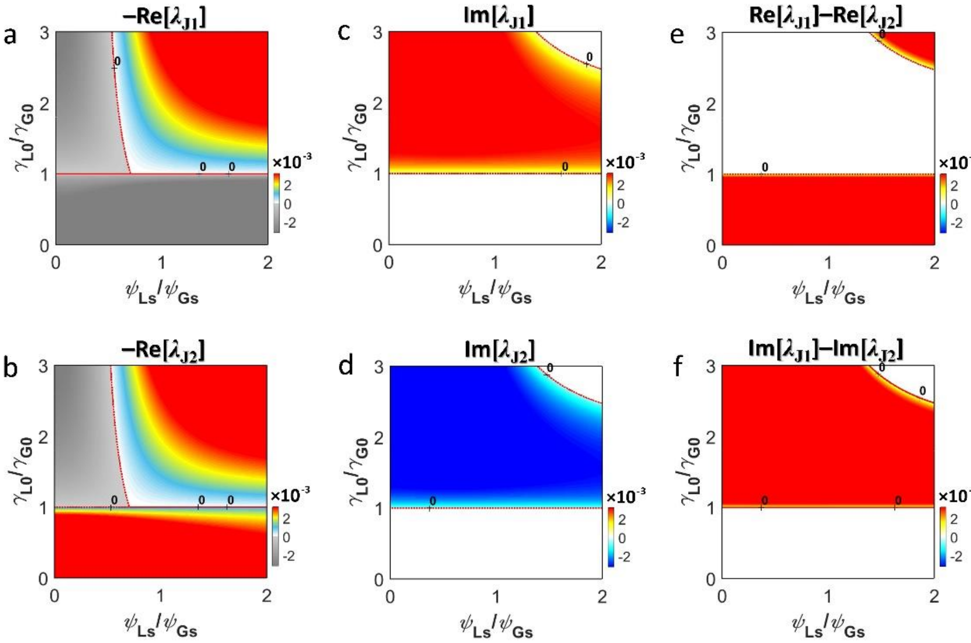

Figure S1. Stability of the equilibria in the broken PT-symmetric phase. (a-d) Real and imaginary components of Jacobian eigenvalues for the classification in PT-symmetric dynamics: (a) –Re[λ_{J1}], (b) –Re[λ_{J2}], (c) Im[λ_{J1}], and (d) Im[λ_{J2}]. (e,f) Differences in each component: (e) Re[λ_{J1}] – Re[λ_{J2}] and (f) Im[λ_{J1}] – Im[λ_{J2}].

Except for the boundary (Re[$\lambda_{J1,J2}$] = 0), the entire regime corresponds to hyperbolic equilibria. From Figure S1, the broken PT-symmetric phase is thus divided into 3 classes (Figure S2a): (b)

unstable equilibrium as the "focus-type" source, (c) stable equilibrium as the "focus-type" sink, and (d) stable equilibrium as the "node-type" sink. The regimes of (b,c) have different stability conditions separated by the 2-dimensional invariant manifold state ($\mathrm{Re}[\lambda_{J1,J2}] = 0$) according to the center manifold theorem. Despite different types of phase portraits, the (c,d) regimes exhibit the same stability condition. Figure S2b-d shows examples of the phase portraits for b-d in Figure S2a.

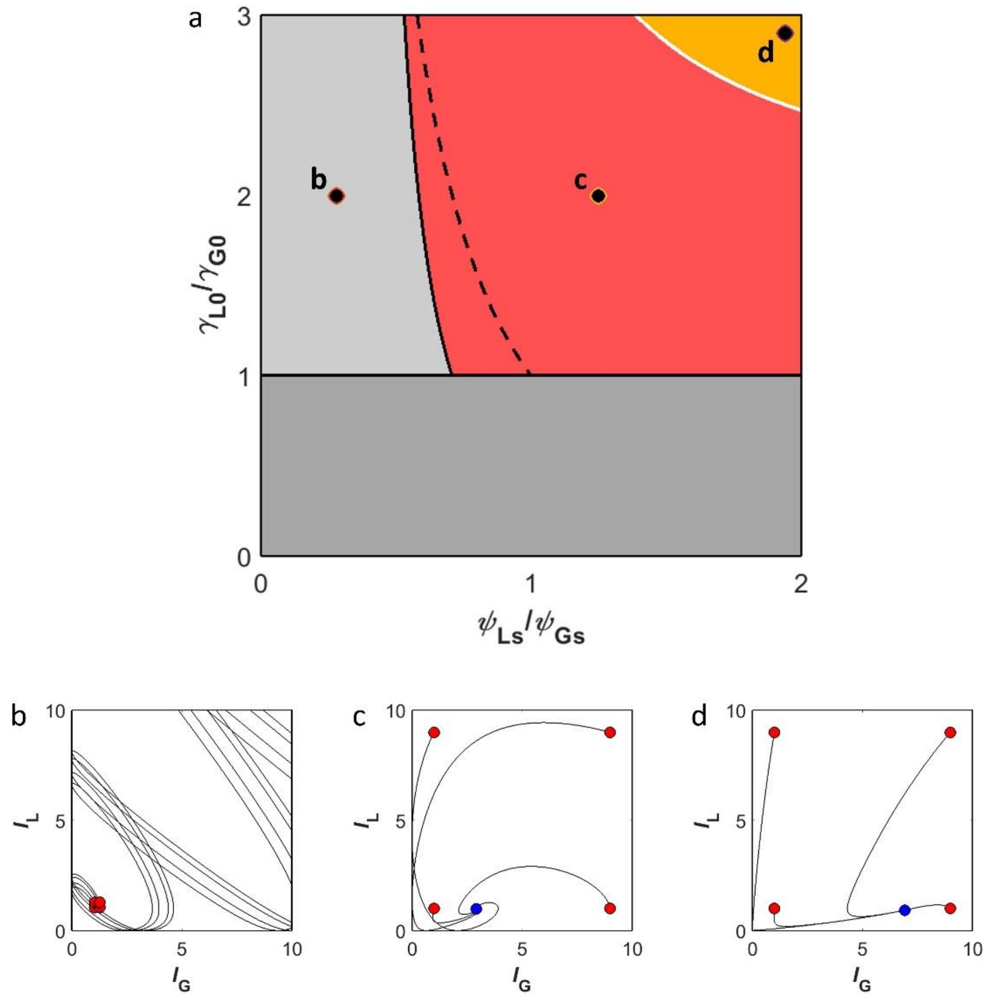

Figure S2. Classification of the broken PT-symmetric phase. (a) Phase diagram defined by the stability of the phase portrait. (b-d) Phase portraits for points b-d in (a). Red (or blue) circles represent the initial (or final) intensity states for a simulation of 10^7 time steps.

Note S3. AD-OD phase transition boundary

The black arrow in Figure 3 in the main text represents the AD-OD transition boundary. While this boundary corresponds to the EP in PT-symmetric phases, the equilibrium intensities (Figure 2a,b in the main text) show that the boundary also meets the convergence of the nontrivial equilibrium to the "trivial" equilibrium $[I_G, I_L] = [0, 0]$. Figure S3 shows examples of phase portraits in this state (point A in Figure 3a in the main text), demonstrating convergence to the trivial equilibrium $[I_G, I_L] = [0, 0]$ independent of the initial intensity or excitation port.

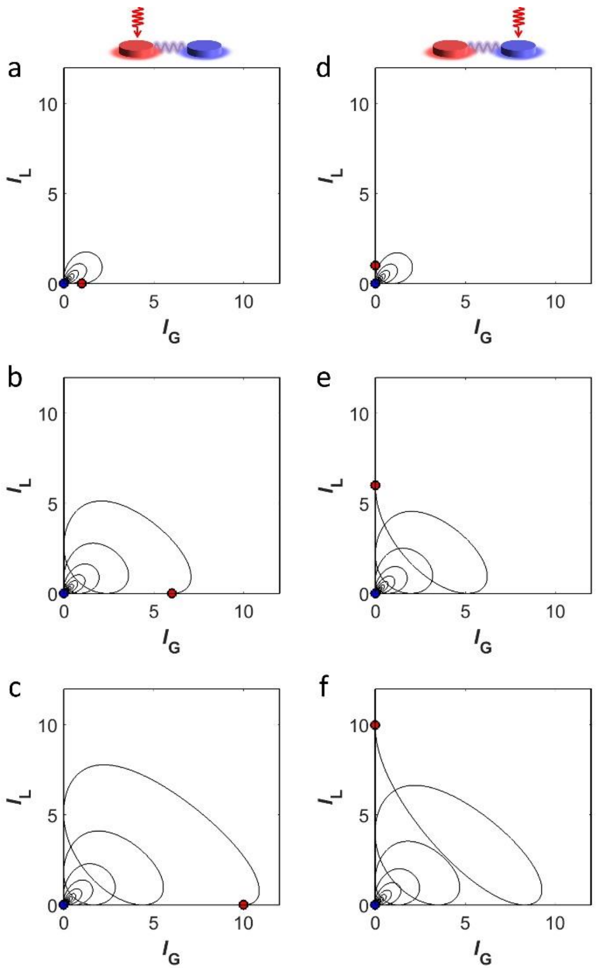

Figure S3. Phase portraits in the I_G-I_L space: (a-c) gain and (d-f) loss resonator excitation for point A in Figure 3a in the main text. The initial light intensities are (a,d) $I = 1$, (b,e) $I = 6$, and (c,f) $I = 10$. Red (or blue) circles represent the initial (or final) intensity states for the simulation of 10^7 time steps.

Note S4. C1 coexisting phase dynamics

The C1 phase in Figure 3a in the main text is defined by the coexistence of (i) the stable equilibrium of the broken PT-symmetric phase and (ii) the unstable equilibrium of the unbroken PT-symmetric phase. Dependent on the initial condition, the steady-state solution can then become OD oscillation quenching due to the stable equilibrium or divergent due to the unstable equilibrium. Figure S4 shows examples of phase portraits in this state (point B in Figure 3a in the main text), exhibiting the OD state (Figure S4a-d) and unstable solutions (Figure S4e,f) dependent on the initial intensity.

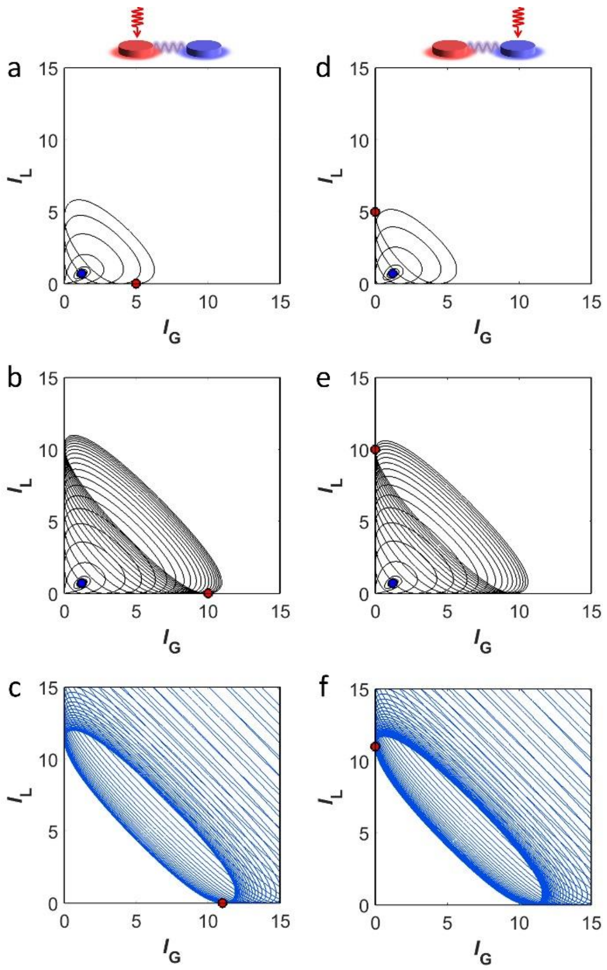

Figure S4. Phase portraits in the I_G-I_L space: (a-c) gain and (d-f) loss resonator excitation for point B in Figure 3a in the main text. The initial light intensities are (a,d) I = 5, (b,e) I = 10, and (c,f) I = 11. Red (or blue) dots represent the initial (or final) intensity states for a simulation of 10^7 time steps. Blue lines in (c,f) denote the divergent loci.

Note S5. Oscillatory PT-symmetric phase transition

To examine the PT-symmetric phase of the C2 coexisting phase, we apply the harmonic approximation of Equation (7) in the Experimental Section 7.2 in the main text. The phase of PT symmetry is then classified by the dynamic indicator $D(t)$: $D(t) > 0$ for the unbroken phase having two eigenfrequencies with different real parts, $D(t) < 0$ for the broken phase having two eigenfrequencies with different imaginary parts, and $D(t) = 0$ for the EP having the eigenfrequencies coalesced to a single complex value.

Figure S5 shows the temporal evolutions of $D(t)$ with gain (Figure S5a-c) and loss (Figure S5d-f) resonator excitations, which are calculated from Equation (7) with the results of the Runge-Kutta-based time-domain analysis for Equation (1) (point C in Figure 3a in the main text). Although the C2 phase supports the unstable equilibria for both unbroken ($D(t) > 0$) and broken ($D(t) < 0$) PT-symmetric phases in the analysis based on the Lyapunov criterion, the oscillation between these phases can lead to stable limit cycles (Figure 5a,d,e in the main text), achieving the repetitive firing of light (Supporting Information Note S6). Figure S5a,d,e demonstrates that this stable state corresponds to the “dynamic oscillation” across the EP ($D(t) = 0$). Because of the oscillatory evolution near the EP, the two resonators are almost synchronized with a fixed phase difference (Supporting Information Note S6), which is explained by the coalescence of eigenmodes with chirality. The numerical quantification of this phase difference is shown in Supporting Information Note S7.

In terms of the modulation response analysis, the temporal variation of $D(t)$ can be divided into large- and small-signal responses. Because the average $D(t)$ of the repetitive firing state is under broken PT symmetry ($D(t) < 0$), the dynamic oscillation across the EP can also be understood as (i) broken PT symmetry for the large-signal response with (ii) the oscillatory modulation for the small-signal response, which becomes the source of the stable limit cycle from the dynamic transition between unbroken and broken PT-symmetric phases.

In contrast to the repetitive firing states (Figure S5a,d,e), the unstable states with the divergence of light intensity correspond to the unstable equilibrium of the unbroken PT symmetry

($D(t)$ > 0 in Figure S5b,c,f). While oscillator behaviors also exist in $D(t)$, these oscillations are damped in the unbroken PT-symmetric phase.

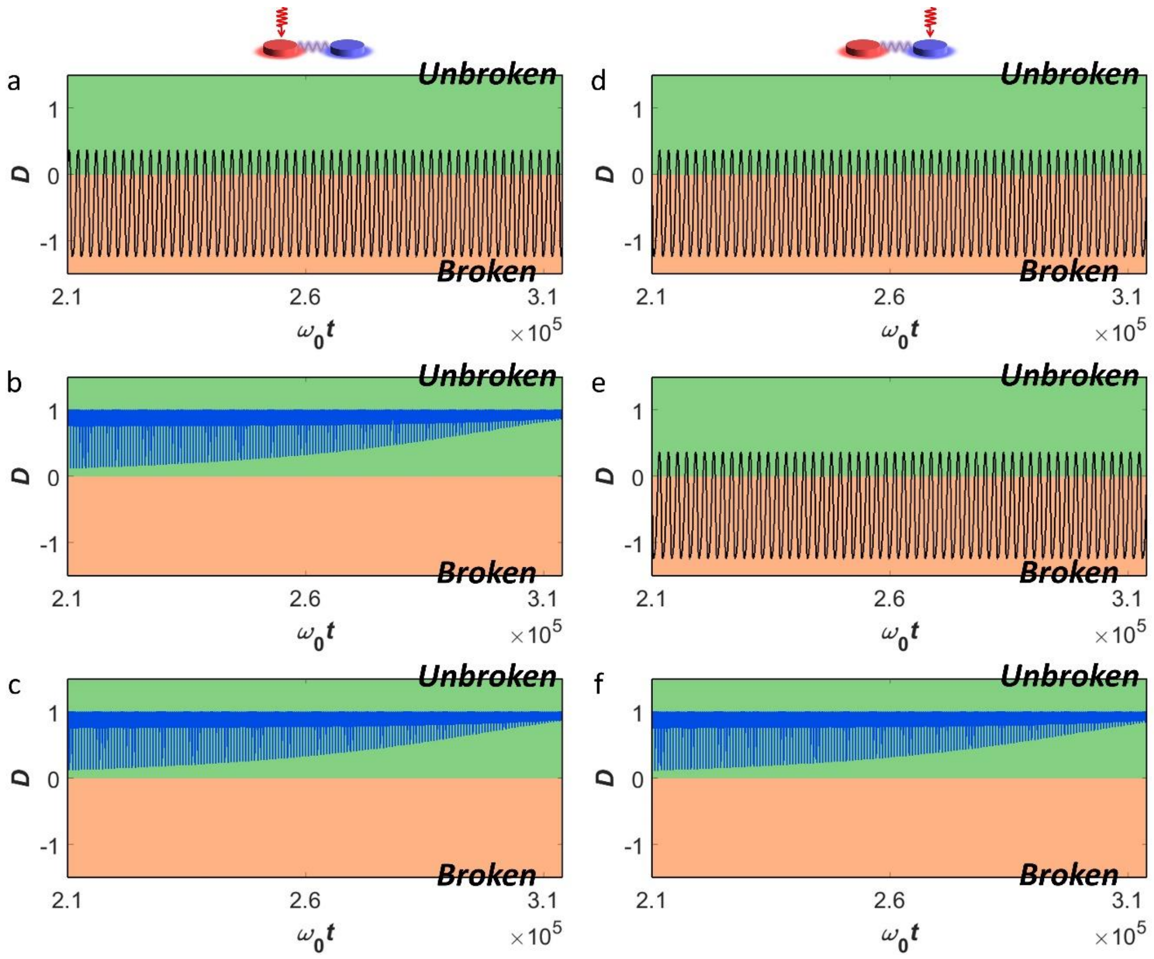

Figure S5. PT symmetry phase indicator $D(t)$: (a-c) gain and (d-f) loss resonator excitation for point C in Figure 3a in the main text. The calculation of $D(t)$ is based on the results of Equation (1) in the main text, solved by the 6th-order Runge-Kutta method. The initial light intensities are (a,d) 0.5, (b,e) 1.2, and (c,f) 1.4. The black lines in (a,d,e) denote the convergent cases of repetitive firing, and the blue lines in (b,c,f) denote the divergent cases. Green (or orange) background color represents unbroken (or broken) PT symmetry.

Note S6. Tunability and directionality in photonic repetitive firing

Figure S6 shows the time-varying evolutions in the C2 phase. Repetitive excitations of light are achieved for initial conditions below the threshold (yellow), similar to the self-pulsation in lasers with a saturable absorber[1], while the light intensity increases for initial conditions above the threshold (aqua). The threshold intensities I_{th} of the gain (Figure S6a) and loss resonator (Figure S6b) excitation differ from each other (I_{th} = 0.82 for gain case and I_{th} = 1.21 for loss case), which imposes directionality on the photonic neuron, an inherent property of PT symmetry.[2-4] Notably, the convergence "speed" to the limit cycle in the repetitive firing is manipulated dependent on the initial intensity (Figure 5d,e in the main text). With identical limit cycles that have different convergence times, the full coverage of the temporal phase of photonic repetitive firing becomes possible (Figure S6a,b).

Furthermore, the signals of gain and loss resonators in repetitive firing are almost in-phase, similar to coupled SL-type oscillators with the designed phase delay.[1, 5, 6] This implies that gain and loss coefficients incorporated with evanescent coupling without any phase delay enable the effective phase delay, as demonstrated in the eigenpolarization evolution in PT-symmetric structures.[7] Temporal phase differences between resonators for both fields and amplitudes are quantified by transforming Equation (1) into coupled real-valued equations (Supporting Information Note S7).

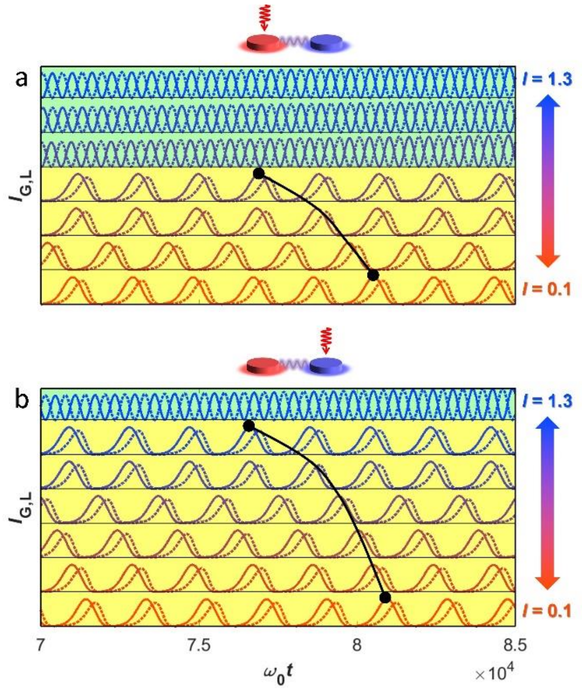

Figure S6. Temporal dynamics of repetitive firing in the photonic neuron for the initial condition of (a) gain and (b) loss resonator excitations. The initial intensity varies as $I = 0.1$ to 1.3. For the 10^7 time step simulation with the unit step $2\pi/(200\omega_0)$, the time domain of $7.0\times10^4 \le \omega_0 t \le 8.5\times10^4$ is shown. The yellow (or aqua) background represents the limit cycle (or unstable) state. The black solid lines show the evolution of the excitation peak, controlled by the excited field intensity.

Note S7. Temporal phase differences in field and amplitude

Following the discussion in Note S6, we examine the temporal phase differences between the signals of gain and loss resonators for both the field and intensity of the signals, which is a useful tool for analyzing neuronal repetitive firing. We set $\psi_G(t) = [I(t)]^{1/2}\cos[\vartheta(t)]\exp[+i\varphi(t)/2]\exp[i\omega(t)]$ and $\psi_L(t) = [I(t)]^{1/2}\sin[\vartheta(t)]\exp[-i\varphi(t)/2]\exp[i\omega(t)]$ with real-valued parameters $I(t)$, $\vartheta(t)$, $\varphi(t)$, and $\omega(t)$, which correspond to the average intensity, the intensity phase, the field phase, and the average frequency. The derivatives of the field amplitudes $\psi_{G,L}$ are then

$$\begin{aligned}\frac{d\psi_G}{dt} &= \left[\frac{1}{2I}\cdot\frac{dI}{dt} - (\tan\theta)\cdot\frac{d\theta}{dt} + \frac{i}{2}\cdot\frac{d\varphi}{dt} + i\frac{d\omega}{dt}\right]\cdot\psi_G \\ \frac{d\psi_L}{dt} &= \left[\frac{1}{2I}\cdot\frac{dI}{dt} + (\cot\theta)\cdot\frac{d\theta}{dt} - \frac{i}{2}\cdot\frac{d\varphi}{dt} + i\frac{d\omega}{dt}\right]\cdot\psi_L\end{aligned}. \qquad \text{(S2)}$$

With Equation (S2), Equation (1) in the main text becomes

$$\begin{aligned}&\left[\frac{1}{2I}\cdot\frac{dI}{dt} - (\tan\theta)\cdot\frac{d\theta}{dt} + \frac{i}{2}\cdot\frac{d\varphi}{dt} + i\frac{d\omega}{dt}\right] - \left(i\omega_0 + \gamma_G\cdot\frac{1}{1+\frac{I\cos^2\theta}{I_G}}\right) = i\kappa\cdot e^{-i\varphi}\cdot\tan\theta \\ &\left[\frac{1}{2I}\cdot\frac{dI}{dt} + (\cot\theta)\cdot\frac{d\theta}{dt} - \frac{i}{2}\cdot\frac{d\varphi}{dt} + i\frac{d\omega}{dt}\right] - \left(i\omega_0 - \gamma_L\cdot\frac{1}{1+\frac{I\sin^2\theta}{I_L}}\right) = i\kappa\cdot e^{i\varphi}\cdot\cot\theta\end{aligned}. \qquad \text{(S3)}$$

Because $I(t)$, $\vartheta(t)$, $\varphi(t)$, and $\omega(t)$ are real, Equation (S3) can be divided into real and imaginary parts as

$$\begin{aligned}&\frac{1}{2I}\cdot\frac{dI}{dt} - (\tan\theta)\cdot\frac{d\theta}{dt} - \gamma_G\cdot\frac{I_G}{I_G + I\cos^2\theta} = \kappa\cdot\tan\theta\cdot\sin\varphi \\ &\frac{1}{2I}\cdot\frac{dI}{dt} + (\cot\theta)\cdot\frac{d\theta}{dt} + \gamma_L\cdot\frac{I_L}{I_L + I\sin^2\theta} = -\kappa\cdot\cot\theta\cdot\sin\varphi \\ &\frac{1}{2}\cdot\frac{d\varphi}{dt} + \frac{d\omega}{dt} - \omega_0 = \kappa\cdot\tan\theta\cdot\cos\varphi \\ &-\frac{1}{2}\cdot\frac{d\varphi}{dt} + \frac{d\omega}{dt} - \omega_0 = \kappa\cdot\cot\theta\cdot\cos\varphi\end{aligned}. \qquad \text{(S4)}$$

Combining the first and second equations in Equation (S4) yields

$$\begin{aligned}\frac{dI}{dt} &= I\cdot\sin 2\theta\cdot\left(\gamma_{G0}\cdot\frac{I_{Gs}\cdot\cot\theta}{I_{Gs}+I\cos^2\theta}-\gamma_{L0}\cdot\frac{I_{Ls}\cdot\tan\theta}{I_{Ls}+I\sin^2\theta}\right)\\ \frac{d\theta}{dt} &= -\kappa\sin\varphi-\frac{\sin 2\theta}{2}\cdot\left(\gamma_{G0}\cdot\frac{I_{Gs}}{I_{Gs}+I\cos^2\theta}+\gamma_{L0}\cdot\frac{I_{Ls}}{I_{Ls}+I\sin^2\theta}\right)\end{aligned}, \quad \text{(S5)}$$

while combining the third and fourth equations of Equation (S4) yields

$$\frac{d\varphi}{dt} = -2\kappa\cdot\cot 2\theta\cdot\cos\varphi, \quad \text{(S6)}$$

with $d\omega/dt = \omega_0 + \kappa\cos(\varphi)/\sin(2\vartheta)$. Equations (S5) and (S6) fully describe the dynamic evolution of optical states in the three-dimensional parameter space (I, ϑ, φ).

Figure S7a,b shows the evolutions of optical states, each for the gain and loss resonator excitation (I = 1 at t = 0). The directional rotation in the ϑ-φ parameter space occurs for both cases. However, depending on the initial excitation channel (red and blue symbols in Figure S7), the bifurcation of the system between the unstable (Figure S7a) and stable limit cycle state (Figure S7b) is evident, representing the "open" (diverging I) and "closed" (conservative I) cycles for each case in the ϑ-I plane.

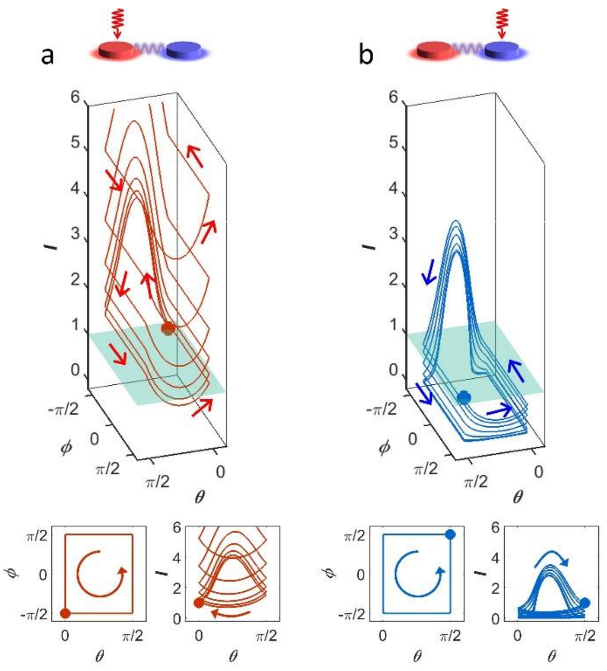

Figure S7. Temporal phase analysis of the intensity and field of signals in gain and loss resonators in the I-ϑ-φ space: (a) gain and (b) loss resonator excitation for point C in Figure 3a in the main text. The initial light intensities are I = 1 for both cases. Bottom figures show the trajectories on the ϑ-φ and ϑ-I planes. Solid circles denote the initial point, and arrows present the direction of the evolution.

References for Supporting Information

[1] J. L. Dubbeldam, B. Krauskopf, *Opt. Commun.* **1999**, *159*, 325.

[2] L. Feng, R. El-Ganainy, L. Ge, *Nat. Photon.* **2017**, *11*, 752.

[3] R. El-Ganainy, K. G. Makris, M. Khajavikhan, Z. H. Musslimani, S. Rotter, D. N. Christodoulides, *Nat. Phys.* **2018**, *14*, 11.

[4] Ş. K. Özdemir, S. Rotter, F. Nori, L. Yang, *Nat. Mater.* **2019**.

[5] C. R. Mirasso, P. V. Carelli, T. Pereira, F. S. Matias, M. Copelli, *Chaos* **2017**, *27*, 114305.

[6] E. Clerkin, S. O'Brien, A. Amann, *Phys. Rev. E* **2014**, *89*, 032919.

[7] S. Yu, H. S. Park, X. Piao, B. Min, N. Park, *Optica* **2016**, *3*, 1025.
